# Immune evolution and therapeutic vulnerabilities in thyroid cancer: from inflammation to immune escape

**DOI:** 10.3389/fimmu.2026.1850228

**Published:** 2026-07-01

**Authors:** Xiaotong Qiu, Li Zhao, Yingli Ding, Yongji Jiang, Simin Liu, Chuanzi Zuo, Yanlei Huo, Zhongwei Lv, Chao Ma

**Affiliations:** 1 Department of Nuclear Medicine, Shanghai Tenth People’s Hospital, School of Medicine, Tongji University, Shanghai, China; 2 Qingdao Central Hospital, University of Health and Rehabilitation Sciences, Qingdao Central Hospital, Qingdao, China; 3 Department of Nuclear Medicine, Shanghai Jiaotong University, Shanghai, China; 4 Shanghai Public Health Clinical Center, Fudan University, Shanghai, China

**Keywords:** immune cell infiltration, immune microenvironment, immune phenotypes, immunotherapy, thyroid cancer

## Abstract

Thyroid cancer exhibits substantial heterogeneity in its tumor immune microenvironment (TIME), which critically shapes disease progression and therapeutic responsiveness. While most differentiated thyroid cancers (DTCs) remain indolent, a subset evolves into radioiodine-refractory disease or progresses to poorly differentiated (PDTC) and anaplastic thyroid carcinoma (ATC), characterized by aggressive behavior and limited treatment options. Emerging evidence suggests that this transition is accompanied by dynamic immune reprogramming rather than static immune evasion. In this review, we propose a stepwise model of immune evolution in thyroid cancer, spanning from autoimmune-driven inflammation in chronic lymphocytic thyroiditis (CLT) to immune-exhausted states in advanced tumors. We systematically characterize immune cell composition, functional states, and regulatory networks across disease stages, highlighting key shifts in antigen presentation, T-cell functionality, and myeloid cell polarization. Building on this framework, we integrate tumor immune phenotypes (“hot”, “altered”, and “cold”) with actionable biomarkers, including PD-L1 expression, tumor mutational burden, IFN-γ signatures, M2 macrophage-related signature, and tertiary lymphoid structures. We further map these immune contexts to rational therapeutic strategies, encompassing immune checkpoint blockade, combination regimens with tyrosine kinase inhibitors or radiotherapy, and emerging approaches such as innate immune activation and adoptive cell therapies. By linking immune evolution with therapeutic vulnerabilities, this review provides a translationally relevant framework for precision immunotherapy in thyroid cancer and highlights future directions for overcoming resistance in advanced disease.

## 1 Introduction

Thyroid cancer (TC), the most common endocrine malignancy, has witnessed a global surge in incidence over recent decades, now ranking as the ninth most common cancer worldwide (1, 2). Most differentiated thyroid cancers (DTCs) are curable with surgery and radioactive iodine (RAI) (3), but a clinically meaningful subset becomes RAI-refractory or progresses to poorly differentiated (PDTC) or anaplastic thyroid carcinoma (ATC), entities notorious for therapeutic resistance and dismal survival (4–6).

Recent advances in tumor immunology have highlighted the immune microenvironment as a critical determinant of TC behavior (7). Chronic lymphocytic thyroiditis (CLT), an autoimmune disorder frequently coexisting with papillary thyroid carcinoma (PTC), provides a unique context for understanding immune activation and suppression in thyroid tissues. Despite the clinical success of immune checkpoint inhibitors (ICIs) in various solid tumors, the response in TC remains heterogeneous and context dependent. However, current studies largely describe immune components in isolation, and a unified framework linking immune evolution with therapeutic vulnerabilities remains lacking.

In this review, we propose an integrative model of immune microenvironment evolution in thyroid cancer, spanning from autoimmune inflammation to immune escape. We further connect immune phenotypes with actionable biomarkers and therapeutic strategies, aiming to provide a clinically relevant framework for precision immunotherapy.

## 2 Immune microenvironmental evolution in thyroid

### 2.1 From autoimmune thyroiditis to thyroid cancer

Inflammation, functioning as the organism’s defensive response to tissue damage, is an essential physiological protective mechanism. Francesco et al. characterized tumor-associated inflammation as the seventh biological feature of cancer (8), a concept corroborated by Douglas Hanahan and Robert A. Weinberg, who identified tumor-promoting inflammation as a critical hallmark of cancer (9). Tumors have been defined as “wounds that do not heal”, a description rooted in the sustained cell renewal and proliferation caused by tumor-associated inflammation (10). In the context of the thyroid, Dailey et al. were the first to propose a correlation between TC and Hashimoto’s thyroiditis (11). CLT, or Hashimoto’s thyroiditis, is an organ-specific autoimmune disorder characterized by follicular cell destruction, germinal center formation, and diffuse infiltration of T and B lymphocytes. The immune microenvironment in CLT is marked by abundant CD4^+^ helper T cells, CD8^+^ cytotoxic T lymphocytes, activated B cells, M1-polarized macrophages, and mature dendritic cells. These immune cells produce interferon-gamma (IFN-γ) and other proinflammatory cytokines, upregulate MHC class II and co-stimulatory molecules, and may elevate immune checkpoint ligand expression, including PD-L1 (12, 13).

CLT frequently coexists with PTC, and this coexistence exerts dual effects on the tumor microenvironment. On one hand, CLT induces heavy lymphocytic infiltration (T cells, B cells, plasma cells), which may enhance immune surveillance. Clinically, PTCs with concurrent CLT exhibit reduced rates of vascular invasion, extrathyroidal extension, recurrence, and mortality (14). The severity of thyroiditis correlates positively with lymphocyte density within tumors, suggesting immune-mediated control of cancer progression (15). On the other hand, chronic inflammation in CLT upregulates immunosuppressive factors. In PTC associated with CLT, key immune checkpoints (e.g. PD-L1) are more highly expressed (16, 17), driven by cytokines such as IL-4/IL-10 that are elevated in the thyroiditis milieu (18). Indeed, PTCs arising in the setting of CLT often show higher PD-L1 levels than those without thyroiditis, which can impair CD8^+^ T-cell cytotoxicity and facilitate immune escape (19). Thus, CLT establishes a dual role: it may restrain tumor progression via enhanced immune activation, yet simultaneously fosters an immunosuppressive niche through checkpoint upregulation and Treg accumulation. Despite this duality, CLT remains a favorable prognostic indicator in PTC (14).

Importantly, not all lymphocyte infiltration reflects protective immunity. In PTCs lacking CLT but showing intratumoral tumor-infiltrating lymphocytes (TILs), disease stage and aggressiveness tend to be higher, especially when dominated by regulatory CD4^+^ T cells rather than CD8^+^ subset (20). This suggests that autoimmune and anti-tumor responses should be distinguished, rather than conflated (20). Thyroid autoimmune diseases and TC are present at opposite ends of the spectrum of immune responses. In TC pathogenesis, the immune response appears tolerant, enabling tumor survival and growth. In contrast, autoimmune thyroid disease triggers a destructive immune response, leading to thyroid function failure.

Beyond its prognostic significance in PTC, CLT may also contribute to thyroid carcinogenesis. Chronic thyroiditis induces transcription factors such as NF-κB, which in turn upregulates matrix metalloproteinases-MMP-3, via CCL20/CCR6 signaling and MMP-9 transcription, thereby promoting thyroid cancer cell invasion and metastasis (21, 22). Inhibition of NF-κB can restore TNF-α–mediated apoptosis in thyroid cancer cells through sustained JNK activation, highlighting a potential immunotherapeutic avenue.

Clinically, the net effect of CLT in PTC is generally favorable. However, its impact on response to immune checkpoint inhibitors remains to be fully elucidated. The coexistence of a rich lymphoid infiltrate and elevated PD-L1 suggests that CLT-associated tumors might be particularly amenable to PD-1/PD-L1 blockade, albeit with a potential increase in immune-related adverse events given the underlying autoimmunity.

### 2.2 Immune reprogramming during the transition from DTC to PDTC and ATC

**Figure 1:**
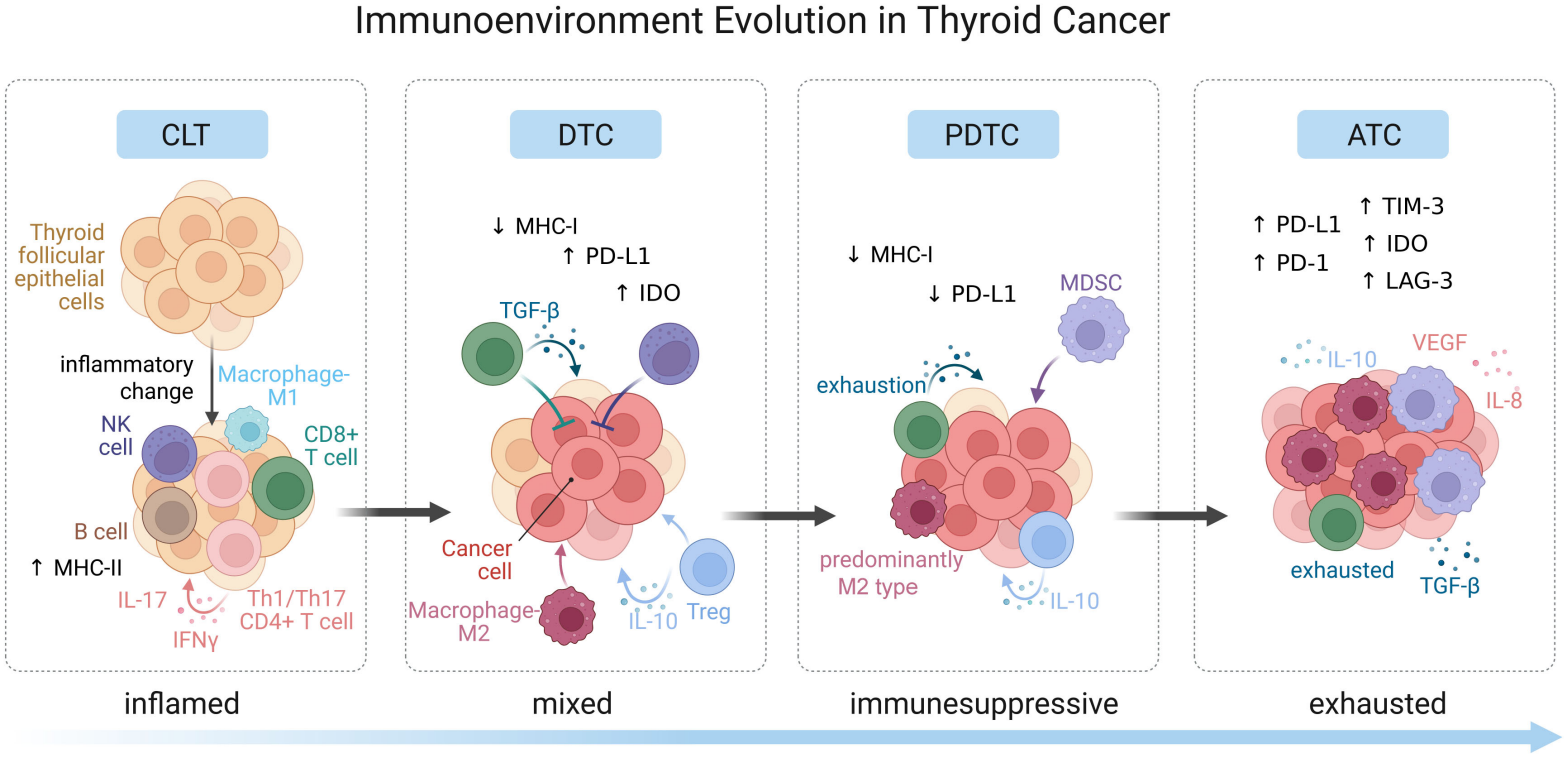
Immune microenvironment evolution across the thyroid cancer continuum. This schematic illustrates the progressive remodeling of the thyroid tumor immune microenvironment, from the pro-inflammatory milieu in chronic lymphocytic thyroiditis (CLT), characterized by abundant CD8^+^ T cells, M1 macrophages, and NK cells, to the increasingly immunosuppressive and exhausted states in differentiated (DTC), poorly differentiated (PDTC), and anaplastic thyroid carcinoma (ATC). Along this trajectory, MHC-I expression declines, immunosuppressive cells such as Tregs, M2 macrophages, and MDSCs increase, and inhibitory ligands (PD-L1, IDO, TIM-3, LAG-3) become upregulated, contributing to tumor immune evasion.

The transition from DTC to PDTC and ATC reflects not only genetic dedifferentiation but also profound immune reprogramming. In DTC, the TME transitions from an inflamed to an altered-immunosuppressive phenotype (23, 24). This is characterized by a decline in antigen presentation, increased infiltration of regulatory T cells (Tregs), functional exhaustion of CD8^+^ T cells, and elevated PD-L1 expression (**Figure 1**). Although some DTCs retain a CLT-like immune infiltrate, their effector functions are often dampened (24–26). Transcriptomic analyses have identified autoimmune-like gene signatures, particularly IFN-γ responsive and B cell related pathways, in subsets of DTC. These findings suggest the presence of intermediate immune states along the spectrum from inflammation to malignancy (26). Immune contexture in this transitional stage may serve as a biomarker for progression risk and therapeutic responsiveness.

With progression to PDTC and ATC, the immune microenvironment undergoes further deterioration (27). PDTCs frequently exhibit an immune-cold phenotype, characterized by low T-cell infiltration, downregulated antigen presentation machinery, and minimal immune checkpoint expression (28). In contrast, ATCs often present as immune-exhausted tumors: although they harbor abundant immune infiltrates, especially tumor-associated macrophages (TAMs), myeloid-derived suppressor cells (MDSCs), and dysfunctional PD-1^+^/CTLA-4^+^ T cells, these cells are ineffectual due to a highly immunosuppressive milieu (29). ATCs shape a highly immunosuppressive microenvironment by secreting cytokines such as TGF-β (30), activating metabolic pathways like indoleamine 2,3-dioxygenase (IDO)-mediated tryptophan catabolism (28), and remodeling stromal and endothelial compartments to impede immune cell infiltration (23). These changes are often accompanied by the accumulation of immunosuppressive cells, including TAMs and MDSCs (31, 32). Therapeutic strategies targeting these mechanisms—such as macrophage repolarization, suppression of immunosuppressive cytokines, and dual immune checkpoint blockade—are emerging as promising approaches in ATC immunotherapy (31).

### 2.3 Tumor–immune communication networks revealed by single-cell technologies

High-resolution single-cell transcriptomics and advanced computational frameworks (e.g., CellChat, CellPhoneDB, and NicheNet) have decoded the complex cellular dialogue within the thyroid tumor immune microenvironment (TIME), moving beyond static compositional descriptions to dynamic ligand-receptor interaction networks (33). During the malignant transition from differentiated thyroid cancer (DTC) to advanced anaplastic thyroid carcinoma (ATC), this communication network shifts from immune-supportive wiring to a highly coordinated immunosuppressive circuit (33). Key oncogenic drivers, particularly the BRAF^V600E^ mutation, orchestrate the secretion of distinct chemokines; for instance, tumor cell-derived CCL20 and CCL20–CCR6 signaling actively recruit regulatory T cells (Tregs) to construct an immune-tolerant niche, a phenomenon that scales with the aggressive dedifferentiation process (34). Concurrently, tumor-associated macrophages (TAMs) engage in dense bidirectional communication with both malignant cells and dysfunctional T cells. Malignant cells secrete colony-stimulating factor 1 (CSF1) to bind CSF1R on TAMs, driving their M2-like polarization and survival (35). In turn, these polarized TAMs release CXCL8 (IL-8) to enhance tumor invasion, alongside transforming growth factor-beta (TGF-β) and interleukin-10 (IL-10), which severely dampen CD8^+^ cytotoxic T cell activity via their respective receptors. Furthermore, inter-cellular checkpoint networks are highly enriched in aggressive phenotypes. Malignant cells and myeloid populations exhibit robust expression of programmed death-ligand 1 (PD-L1) and poliovirus receptor (PVR), which interact with receptor programmed cell death 1 (PD-1) and T-cell immunoreceptor with Ig and ITIM domains (TIGIT) on infiltrating lymphocytes, respectively (36). This dense, multi-layered molecular crosstalk actively drives severe lymphocytic exhaustion and therapeutic resistance, identifying these precise ligand-receptor pairs as crucial targets for rational combination immunotherapies.

## 3 Immune cell composition and function in the thyroid tumor microenvironment

### 3.1 CD8^+^ cytotoxic T lymphocytes

Lymphocyte density is related to improved overall survival and reduced recurrence in human PTC (37). Actively proliferating lymphocytes could be a potential predictor of prolonged DFS in children and young adults (38, 39). However, this contradicts the discussion on more advanced tumor disease in lymphocyte-infiltrated tumors (20), necessitating further exploration of the lymphocyte subtypes driving these findings.

CD8^+^ T lymphocytes are essential for immunotherapy, as they dominate tumor destruction (40). Hyperfiltration of CD8^+^ T lymphocytes are associated with improved DFS as a broad immunohistochemical feature of immune cell infiltration in patients with chronic lymphocytic thyroiditis coexisting with DTC (41). Conversely, in another study, CD8^+^ T lymphocyte enrichment was correlated with a higher relapse risk (42). Moreover, major patients with CD8^+^ TIL infiltration (68%) lacked granzyme B^+^ TIL, indicating an impairment in the effectiveness of CD8^+^ TIL (42). CD8^+^ CTLs employ the Fas-FasL and perforin-granzyme pathways to eradicate target cells. Therefore, quantification of CD8^+^ granzyme B^+^ T cells could serve as a high-resolution biomarker of a favorable prognosis in TC. The cytolytic protein granzymes and perforin are regulated by IL-2 and IL-15 (43, 44). It could thus be possible to activate CTLs by increasing IL-2/IL-15 expression in the TC microenvironment or by delivering IL-2/IL-15 to the TC microenvironment. In ATC, although CD8^+^ cells infiltrate, they often express exhaustion markers. Overall, effective CD8^+^ immunity in TC likely requires not just infiltration but also an activating microenvironment (e.g. IL-2) and absence of checkpoints.

Recent single-cell transcriptomic studies have revealed that CD8^+^ T cell populations in thyroid cancer are highly heterogeneous and encompass multiple differentiation states beyond the conventional effector–exhausted paradigm (45). In addition to terminally exhausted CD8^+^ T cells characterized by high expression of PDCD1, LAG3, HAVCR2, and TIGIT, a distinct population of progenitor exhausted T cells (Tpex) has been identified (46–48). These cells retain self-renewal capacity and exhibit responsiveness to immune checkpoint blockade, serving as a critical reservoir for reinvigorated anti-tumor immunity. Furthermore, stem-like CD8^+^ T cells expressing TCF7 and IL-7R have been observed in immune-active thyroid tumors and are associated with sustained immune surveillance and favorable therapeutic responsiveness (49, 50). In contrast, progressive dedifferentiation toward ATC is accompanied by expansion of terminally exhausted CD8^+^ T cells and contraction of stem-like populations, suggesting that the balance between these subsets may influence both disease progression and immunotherapy efficacy (45).

To avoid the toxic effects of systemic IL-2 administration, novel methods of administering IL-2 specifically into tumors have been tested; for instance, attaching IL-2 to tumor-associated ligands or expressing it in other oncolytic viruses. However, a double-edged role of IL-2 in regulating tumor-infiltrating CD8^+^ T cells has been reported, which promotes CD8^+^ T cell activation and proliferation in early tumor initiation, but induces CD8^+^ T cell exhaustion at an advanced tumor stage (43).

### 3.2 CD4^+^ T helper cells (Th1, Th2, Th17, Tfh)

**Figure 2:**
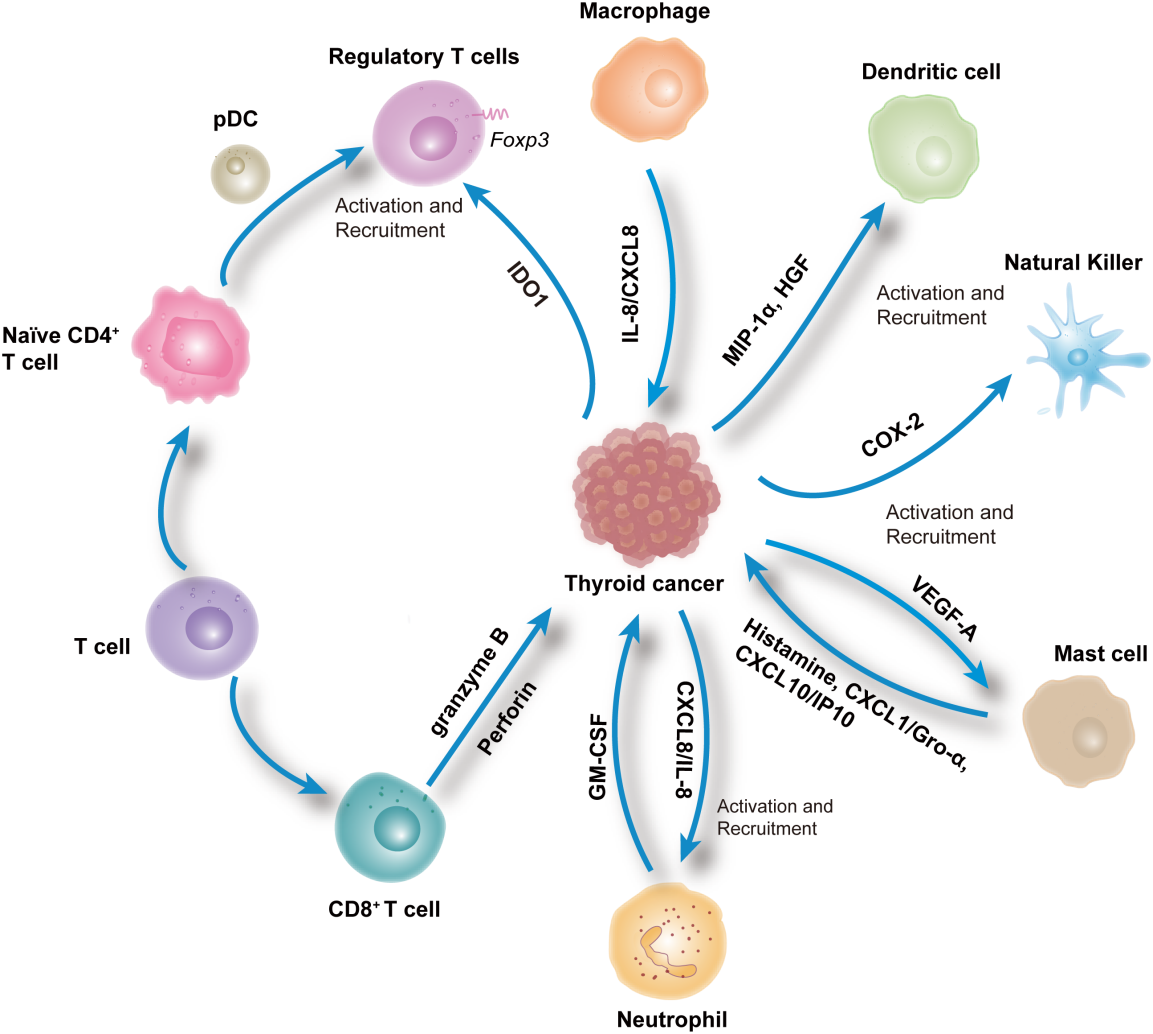
Interactions between cancer cells and immune cells in thyroid cancer. Macrophages play a significant role in promoting tumor progression through the production of CXCL8 and IL-8, which recruit neutrophils that produce GM-CSF involved in tumor progression. VEGF-A recruits mast cells that produce histamine, CXCL1/GRO-α, and CXCL10/IP-10 to stimulate cancer cell proliferation. Furthermore, HGF and MIP-1a facilitate the recruitment of dendritic cells, while thyroid cancer cells produce COX-2 to recruit NK cells. T cells are categorized as CD8^+^ and CD4^+^ based on differentiation antigens. CD8^+^ cells elease granzyme B and perforin to aid thyroid cancer growth. Naïve CD4^+^ T cells transform into FoxP3+ Treg cells with pDCs, while thyroid cancer cells hinder anti-tumor immunity by attracting FoxP3+ T cells through IDO1. pDC, plasmacytoid dendritic cells.

Liu et al. (51) observed that patients with PTC had significantly more CD4^+^ CD25^+^ CD127^low/-^ Tregs than patients with MNG in their peripheral blood, which was linked to extrathyroidal invasion and lymphatic metastasis. Tregs are common in the metastatic lymph nodes of PTC and are associated with recurrent PTC (52). Plasmacytoid DCs (pDCs) in PTC differentiate naive CD4^+^ T cells into FoxP3^+^ ICOS^+^ Tregs through inducible costimulatory ligand (ICOSL), resulting in Treg accumulation in PTC (53). Additionally, the presence of FoxP3^+^ Treg cells is linked to the aggressive characteristics of PTC microcarcinomas. This is due to indoleamine 2,3-dioxygenase1 (IDO1) in tumor cells, which promotes FoxP3^+^ Treg activation and suppresses anti-tumor immunity (54) (**Figure 2**). In addition, in an IHC study of the relationship between FoxP3 lymphocytes and DTC prognosis, tumor size and FoxP3 expression were negatively correlated, and FoxP3 expression influenced DTC aggressiveness (55). Consequently, FoxP3 may predict TC prognosis and serve as a biological target for immunotherapy.

Few studies have investigated the roles of follicular helper T (Tfh)and T helper 17 (Th17) cells in TC. Among CD4^+^ T-cell subsets, Tfh cells have recently emerged as important regulators of anti-tumor immunity in thyroid cancer. Tfh cells support B-cell activation, affinity maturation, and tertiary lymphoid structure (TLS) formation through secretion of IL-21 and expression of CXCR5 (34). Single-cell analyses have identified Tfh-like populations enriched in immune-active papillary thyroid carcinomas, particularly those containing TLS (56). The presence of Tfh cells is frequently associated with increased B-cell infiltration, enhanced antigen presentation, and improved disease-free survival (57). These observations suggest that Tfh cells may contribute to productive anti-tumor immune responses and potentially enhance responsiveness to immunotherapy.

Thyroid tumors have significantly more CD4^+^ IL17^+^ T cells (Th17) than benign thyroid tissue, and the density of Th17 cells is much higher in thyroid tumors than in healthy thyroid tissue. A correlation was found between the density of Th17 in the peripheral blood and the level of IL-17 in the serum (58). In contrast to patients with autoimmune thyroid disorders, T lymphocyte frequency was higher in patients with TC (59). These cells, which express neither CD4 nor CD8, are termed double-negative T (DNT) cells, suppressing T cell growth activation, and subsequently reducing cytokine production in the TME (60). However, double-negative T cells have been shown to enhance immunity in various other conditions, including infiltration in thyroiditis due to immunotherapy (61), which is usually associated with better survival in patients with other cancers being treated with ICIs.

### 3.3 Tumor associated macrophages

TAMs are positively correlated with de-differentiation and unfavorable prognosis in TC (62). TAM density varies across different pathological subsets of TC (63). In a study evaluating TAM in 14 cancers including thyroid, lung, and breast cancers, ATCs exhibit the highest TAM count in TC, which correlates with a worse prognosis, but there was no significant difference in CD163^+^ macrophage density in TNM stage of lung, breast, or thyroid cancers in this study (63). Whereas in other studies, although low TAM counts are typical in patients with PTC, the presence of CD163^+^ M2-type macrophages in patients with PTC correlates with clinical manifestations like larger tumor size, lymph node metastases, and reduced survival (63, 64). This contradictory result may be due to different study designs and sample characteristics. Diffuse sclerosing papillary thyroid carcinoma (DSV-PTC) variant shows M2 macrophages in lymph plugs associated with lymphatic invasion (65). According to Fang et al. (32), TAMs promote PTC invasiveness by secreting IL-8/CXCL8 *in vitro*. Human PTC cells treated with IL-8/CXCL8 metastasized more in immunodeficient mice, confirming this mechanism (32). Paradoxically, in a retrospective analysis of DTC patients, Cunha et al. (41) found a positive correlation between macrophage number and disease-free survival (DFS). Further studies are needed to confirm these conflicting results. Moreover, the enrichment of M1-type macrophages in lung and breast cancers usually contributes to a better clinical prognosis (66, 67). Hence, a further distinction between M1 and M2 macrophages should be made in subsequent studies on TAMs in TC. Recent single-cell studies suggest that the traditional M1/M2 classification may oversimplify macrophage heterogeneity in thyroid cancer (45). Distinct macrophage subsets have been identified based on transcriptional programs and functional properties (68). SPP1^+^ macrophages are enriched in advanced tumors and are associated with extracellular matrix remodeling, angiogenesis, and T-cell dysfunction (68, 69). APOE^+^ macrophages display immunosuppressive characteristics and participate in lipid metabolic reprogramming within the tumor microenvironment (70). In addition, CCL2^+^ macrophages contribute to the recruitment of monocytes and regulatory immune populations, thereby reinforcing chronic immunosuppression (71). These findings suggest that macrophage-targeted therapies should increasingly focus on specific functional subsets rather than the conventional M1/M2 paradigm (45, 68).

Emerging evidence suggests that immunometabolic reprogramming contributes substantially to macrophage function in thyroid cancer. APOE^+^ macrophages exhibit lipid metabolism associated transcriptional programs, whereas hypoxia-driven lactate accumulation may further reinforce immunosuppressive macrophage polarization and T cell dysfunction. In addition, mitochondrial metabolic adaptation has recently been implicated in tumor immune interactions, highlighting immunometabolism as a promising therapeutic target requiring further investigation (72, 73).

### 3.4 B cells, tertiary lymphoid structures, and dendritic cells

Although T-cell-mediated immunity has traditionally dominated thyroid cancer immunology research, increasing evidence suggests that B cells are also important components of the thyroid tumor microenvironment. Single-cell transcriptomic analyses have demonstrated substantial heterogeneity among intratumoral B-cell populations, including naïve B cells, memory B cells, plasma cells, and regulatory B cells (Bregs) (56, 57). In papillary thyroid carcinoma (PTC), increased infiltration of B cells is frequently observed in tumors coexisting with chronic lymphocytic thyroiditis (CLT), where they may contribute to antigen presentation, antibody production, and local immune activation (57).

Recent single-cell studies further suggest that B-cell-rich immune niches are associated with a more indolent tumor phenotype and enhanced anti-tumor immunity. Li et al. identified distinct tumor-infiltrating B-cell subsets associated with favorable prognosis and reduced metastatic potential in PTC, highlighting a previously underappreciated role of B cells in thyroid cancer immune surveillance (56).

Not all B-cell populations exert anti-tumor functions. Regulatory B cells (Bregs), characterized by the production of immunosuppressive cytokines such as IL-10, have been reported in differentiated thyroid cancer (DTC) (74). These cells suppress effector T-cell activation and cytokine secretion while promoting immune tolerance within the tumor microenvironment. Although Bregs may enhance T-cell survival under certain conditions, their overall impact appears to favor immune evasion and tumor persistence (74). The balance between effector B cells and Bregs may therefore represent an important determinant of immune responsiveness in thyroid cancer.

One of the most important recent advances in thyroid cancer immunology is the recognition of tertiary lymphoid structures (TLS). TLS are ectopic lymphoid aggregates composed of B-cell follicles, T-cell zones, mature dendritic cells, and specialized stromal elements that resemble secondary lymphoid organs (34). They frequently arise in chronically inflamed tissues and tumors as a consequence of sustained antigenic stimulation.

In thyroid cancer, TLS are particularly enriched in PTCs associated with Hashimoto’s thyroiditis and represent highly organized immune microenvironments capable of supporting local antigen presentation, B-cell maturation, and T-cell activation (34, 57). Their presence suggests that anti-tumor immune responses may be generated directly within the tumor rather than solely in regional lymph nodes.

Accumulating evidence indicates that TLS are associated with favorable clinical outcomes in thyroid cancer. Li et al. demonstrated that patients with TLS-high PTC exhibited significantly prolonged disease-free survival compared with TLS-low tumors (34). Mechanistically, TLS may enhance anti-tumor immunity through coordinated interactions among B cells, dendritic cells, and effector T cells, thereby promoting immune surveillance and limiting tumor progression.

Consequently, TLS are increasingly recognized not only as prognostic biomarkers but also as potential indicators of immune responsiveness. Similar observations have been reported in melanoma, non-small cell lung cancer, and sarcoma, where TLS-rich tumors demonstrate superior responses to ICIs (75, 76).

Although direct evidence in thyroid cancer remains limited, the emerging role of TLS in other solid tumors suggests potential relevance for immunotherapy stratification. Mature TLS have been associated with increased response rates to PD-1/PD-L1 blockade and improved survival across multiple malignancies. Therefore, incorporating TLS assessment into future thyroid cancer clinical trials may facilitate the identification of patients most likely to benefit from immunotherapeutic interventions.

Dendritic cells (DCs) are professional antigen-presenting cells that play a central role in initiating anti-tumor immunity. In PTC, DC recruitment is promoted through HGF/MET signaling, which induces macrophage inflammatory protein-1α (MIP-1α) secretion and subsequent chemotaxis of CCR6-positive DCs (77). Increased infiltration of S100^+^, CD1a^+^, and CD83^+^ DC populations has been reported in PTC compared with normal thyroid tissue (78).

Importantly, distinct DC subsets appear to exert different biological effects. CD1a^+^ DC density has been associated with improved disease-free survival, whereas S100^+^ DC infiltration shows no clear prognostic significance (78, 79). Conversely, plasmacytoid dendritic cells may contribute to immune escape by promoting differentiation of naïve CD4^+^ T cells into FoxP3^+^ regulatory T cells through ICOSL-mediated signaling (53). Therefore, therapeutic strategies aimed at restoring DC antigen-presenting function or disrupting DC–Treg interactions may represent promising approaches for enhancing anti-tumor immunity in thyroid cancer (80).

### 3.5 Innate effectors: NK cells, tumor associated neutrophils, and mast cells

PTC shows robust tumor-infiltrating NK cells compared to multinodular goiter (MNG) and healthy thyroid tissue. There is a negative correlation between NK cell infiltration and disease stage, suggesting that these cells play a regulatory role in the TC microenvironment (81). ATC cell lines are sensitive to NK cell-mediated lysis *in vitro*, indicating that immunotherapy that recruits activated NK cells to the tumor microenvironment may effectively treat ATC (82). However, tumor cells can evade NK cell-mediated cell lysis by NK cell dysfunction via the PD-1 and T cell immunoglobulin domain and mucin domain (TIM)-3 pathway (83), and by downregulating NKG2D on NK cells through elevated cyclooxygenase (COX) 2 in ATC (82).

The peripheral blood neutrophil-to-lymphocyte ratio (NLR) is commonly explored as a prognostic indicator in TC research. A high NLR is correlated with larger tumors, increased recurrence risk, and more aggressive pathological subtypes (e.g., PDTC and ATC) (84). Patients with TC reported an association between NLR and treatment response. One hundred fifty-one patients with TC were evaluated in this study, and those with limited recurrence risk, stage I diagnosis, and favorable response presented a significantly decreased NLR after treatment. Conversely, the NLR increased in the presence of a poor treatment response. However, it has been argued that the NLR cannot distinguish benign from malignant nodules or predict disease prognosis (85). CXCL8 and IL-8 release by T cells promotes the recruitment of neutrophils, which secrete GM-CSF to support tumor growth. In a study of human TC tissue, TAN density and tumor size were correlated (86).

MCs have been extensively studied in PTC, with a higher density reported in patients with PTC compared to NT tissues and they are associated with tumor growth and invasion (87). Moreover, compared to adenomas, FTCs exhibit higher expression levels of MCs in both intratumoral and peritumoral regions. Therefore, MC density serves as a crucial determinant for distinguishing between benign and malignant follicular thyroid lesions (77). *In vitro* studies of PTC cell lines showed that PTC induces MC chemotaxis through VEGF-A release (87), activating VEGF receptors (VEGFR) on human MCs. Thus, PTC may recruit TMCs via the VEGF pathway. DTC cells activate MCs, releasing cytokines including IL-6, TNF-β, granulocyte colony-stimulating factor (GM-CSF), and chemokines like CXC motif ligand 1/growth regulated oncogene-α (CXCL1/GRO-α), CXCL8/IL-8, and CXCL10/interferon-inducible protein (IP)-10 (41). Sequentially, MCs stimulate TC cell proliferation by producing CXCL1/GRO-α and CXCL10/IP10 (88). Angiogenesis and tumor growth were promoted by recruiting MCs to tumor sites in a mouse model of PTC xenografts. However, these effects were suppressed by MC inhibitors (87). Furthermore, Vichano et al. (89) found that MCs induce epithelial-mesenchymal transition (EMT) in TC cells via CXCL8/IL-8 secretion, maintain the invasive and stem cell properties of TC cells, and demonstrated that the MC-dependent IL-8-Akt-Slug pathway maintains the EMT/stem cell properties of TC cells. In view of this, blocking this pathway emerges as a novel treatment strategy for advanced thyroid cancer.

## 4 Tumor immune phenotypes and clinical implications

### 4.1 Hot, altered, and cold immune phenotypes in thyroid cancer

**Figure 3:**
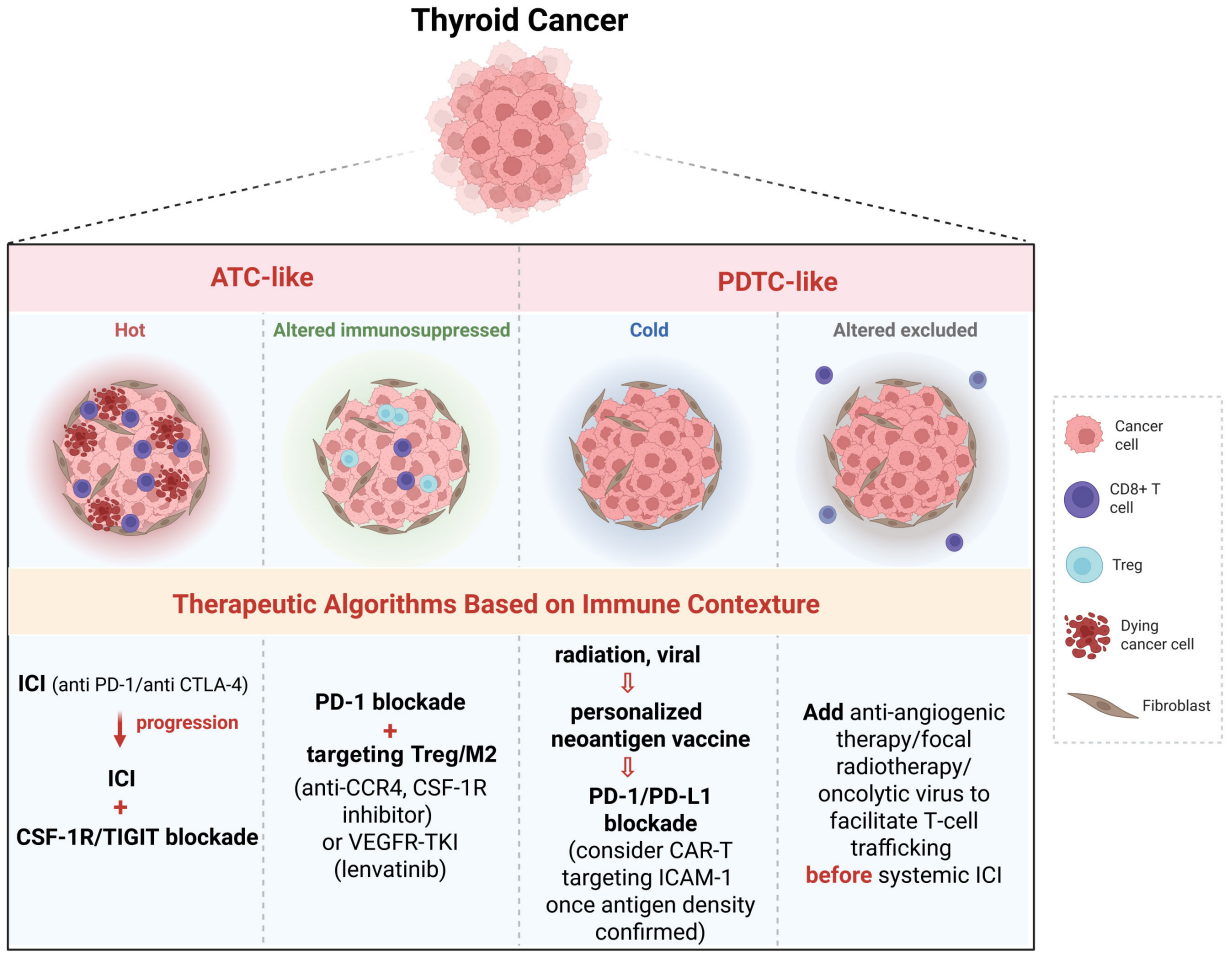
A novel classification method for thyroid cancer based on immunophenotypic characteristics and potential therapeutic strategies. This diagram stratifies thyroid tumors into four immunophenotypic categories, hot (ATC-like), altered immunosuppressed, cold, and altered excluded (PDTC-like), based on immune infiltration and functional status. For hot tumors, initial PD-1/CTLA-4 blockade may be effective, while progression warrants combinatorial strategies (e.g., CSF-1R or TIGIT inhibitors). In altered immunosuppressed tumors, therapies targeting Tregs or M2 macrophages are prioritized. Cold tumors require immune priming via radiation, viral therapies, or personalized neoantigen vaccines before PD-1 blockade. Altered excluded tumors benefit from stromal modulation (e.g., anti-angiogenics or focal radiotherapy) to enhance T-cell trafficking prior to ICI. This immunologic framework offers a rationale for precision immunotherapy in advanced thyroid cancer.

Integrative transcriptomic studies classify thyroid tumors into three broad immune phenotypes, mirroring frameworks used in melanoma and lung cancer (27, 29). Hot (T-cell-inflamed) tumors are rich in effector T cells, dendritic cells, and proinflammatory cytokines, and often express immune checkpoints as an adaptive resistance. ATCs and some DTCs (especially BRAF-mutant PTC) fall into this category, with high infiltration by CD8^+^ T cells and macrophages (90). These tumors also upregulate multiple inhibitory checkpoints (PD-L1/PD-1, LAG-3, TIM-3, TIGIT) (90). Cold (non-inflamed) tumors lack significant immune infiltrates and have “immune ignorance” or exclusion. Many PDTCs and some indolent PTCs are cold, with immune profiles are similar to normal thyroid (90). Altered phenotypes lie between, including “immune-excluded” (immune cells at tumor margin) and “immunosuppressed” (infiltrated but dysfunctional). Giannini et al. identified “altered-excluded” and “altered-immunosuppressed” subsets: the former (seen in many PDTCs) where immune cells surround but do not penetrate tumor nests, and the latter (seen in ATC-like tumors) where T cells are present but exhausted (90). These immune phenotypes have therapeutic implications (**Figure 3**). Hot tumors may respond to checkpoint blockade alone (91), cold tumors likely require combination strategies to “warm” them (e.g. oncolytic viruses, radiation, vaccines to initiate T-cell infiltration) (92), and altered tumors may need both stimulatory (e.g. TLR agonists, cytokines) and inhibitory (checkpoint blockade) interventions to restore immunity (93).

An additional area of interest is indolent papillary thyroid carcinoma (PTC), which may represent a unique state of tumor–immune equilibrium. Unlike aggressive thyroid cancers characterized by immune exhaustion and immunosuppression, indolent PTCs often exhibit limited genomic instability and relatively balanced immune infiltration. These tumors may provide a valuable model for investigating mechanisms of immune surveillance, tumor dormancy, and long-term immune control. Future studies integrating longitudinal clinical observation with spatial immune profiling may help clarify how immune homeostasis contributes to the remarkably favorable outcomes observed in many low-risk PTCs (94).

### 4.2 Actionable immune biomarkers and their clinical implications

As immunotherapy enters the landscape of thyroid cancer treatment, the ability to stratify patients based on immune-related biomarkers has become increasingly important. Several candidate markers derived from tumor cells, immune infiltrates, or transcriptomic features have shown potential for predicting immune responsiveness, guiding combination strategies, and identifying immunologically targetable subtypes. Among these, five biomarker classes stand out for their relative measurability, mechanistic relevance, and clinical actionability:PD-L1 expression (95), Tumor mutational burden (TMB) (96), IIFN-γ gene signature (97), M2 macrophage score (97), and Tertiary lymphoid structures (TLS) (34). Each of these markers is supported by growing evidence in thyroid cancer or across solid tumors, and together they form a preliminary framework for immune stratification (**Table 1**).

**Table 1:** Key characteristics of the actionable immune biomarkers, including detection methods, predictive implications, and disease specific relevance.

Biomarker	Measurement	Clinical implication & stratification	Evidence & future directions in thyroid cancer
PD-L1 expression	IHC: TPS or CPS	Gold standard for identifying “hot” tumors; CPS ≥ 50% serves as a threshold for ICI monotherapy in ATC/PDTC.	Spartalizumab responders cluster in high CPS groups; PD-L1–negative tumors did not respond (95).
TMB	WES or targeted panels (mut/Mb)	Predicts neoantigen load; TMB-high (≥10 mut/Mb) warrants consideration of ICI regardless of PD-L1 status.	Median TMB in ATC ≈ 5-7; responders tend to cluster in upper quartile (96).
IFN-γ gene signature	RNA-seq or PCR analysis of 6-gene or 18-gene IFN-γ response panel	Reflects T-cell inflamed TME; essential for differentiating “warm” from “immunosuppressed” niches.	TCGA analysis identifies an immunologically active cluster (C2) highly enriched for IFN-γ responsive genes with elevated predicted ICI efficacy (97).
M2 TAM score	CD163/CD68 ratio (IHC or RNA)	Key resistance marker; high scores justify combining ICIs with CSF-1R or VEGF inhibitors.	Enriched in aggressive ATC and RAIR-DTC niches; dense macrophage infiltration correlates with profound T-cell exhaustion and poor OS (98).
TLS	IF/IHC detection of CXCL13, LAMP3, PNAd, or gene markers	Superior predictor of OS; mature TLS presence indicates a permissive TME for B cell and T cell synergy.	High TLS density significantly correlates with improved DFS and active regional humoral immunity in PTC cohorts (34).

ATC, anaplastic thyroid carcinoma; CPS, Combined Positive Score; DFS, disease-free survival; DTC, differentiated thyroid cancer; ICI, immune checkpoint inhibitor; IF, immunofluorescence; IHC, immunohistochemistry; Mb, megabase; mut, mutations; OS, overall survival; PDTC, poorly differentiated thyroid cancer; PTC, papillary thyroid carcinoma; RAIR, radioiodine-refractory; TAM, tumor-associated macrophage; TCGA, The Cancer Genome Atlas; TLS, tertiary lymphoid structures; TME, tumor immune microenvironment; TMB, tumor mutation burden; TPS, Tumor Proportion Score; WES, whole-exome sequencing.

Despite their potential utility, currently available immune biomarkers remain imperfect. PD-L1 expression is influenced by antibody selection, scoring algorithms, and intratumoral heterogeneity, leading to inconsistent predictive performance across studies (98). Similarly, TMB is generally low in differentiated thyroid cancer and lacks validated threshold values specific to thyroid malignancies (99). IFN-γ-related signatures may better reflect ongoing immune activation but remain vulnerable to dynamic temporal changes within the tumor microenvironment (100). Therefore, future biomarker development will likely require integrated approaches combining molecular, cellular, and spatial information rather than reliance on any single marker.

Recent advances in single-cell RNA sequencing (scRNA-seq) and spatial transcriptomics have revealed previously unrecognized layers of immune heterogeneity in thyroid cancer (56, 57). Unlike conventional bulk sequencing approaches, these technologies enable simultaneous characterization of immune cell phenotypes, functional states, and spatial organization within the tumor microenvironment (101).

Several emerging biomarkers have recently attracted attention. Exhausted CD8^+^ T-cell populations, particularly progenitor exhausted and stem-like subsets, may identify tumors retaining responsiveness to immune checkpoint blockade (47, 102). Likewise, distinct macrophage programs, including SPP1^+^, APOE^+^, and CCL2^+^ tumor-associated macrophages, have been linked to immune suppression, extracellular matrix remodeling, and tumor progression (68, 103).

Beyond individual cell populations, spatial transcriptomic analyses suggest that the relative positioning of immune cells may carry important biological information (101). For example, close spatial interactions between exhausted T cells and immunosuppressive macrophages may indicate resistance to immune checkpoint inhibitors (104), whereas organized tertiary lymphoid structures may reflect active local immune responses and favorable therapeutic outcomes (34, 76).

Collectively, these findings suggest that future immune stratification in thyroid cancer may evolve from single-marker assessment toward integrated spatial immune ecosystem profiling (101, 104).

## 5 Immunotherapy in thyroid cancer: current status and challenges

### 5.1 Immune checkpoint inhibitors

Immune checkpoint blockade, particularly anti–PD-1/PD-L1 and anti–CTLA-4 therapy, has demonstrated durable responses in multiple solid tumors. However, its efficacy in DTC has been modest, primarily due to low TMB, low neoantigen load, and a relatively “cold” immune microenvironment (105). Despite this, clinical trials of ICIs in TC have yielded mostly modest results. Anti-PD-1 antibodies (pembrolizumab, nivolumab) and anti-CTLA-4 (ipilimumab) have been tested in small cohorts of advanced thyroid cancer. A phase II KEYNOTE-158 trial evaluating pembrolizumab monotherapy in advanced PTC reported an objective response rate (ORR) of only 6.8% (91). Similarly, the efficacy of ICIs in poorly differentiated (PDTC) and anaplastic thyroid carcinoma (ATC) varies greatly depending on tumor immune contexture.

In ATC, despite its aggressive phenotype, there is higher PD-L1 expression and more abundant CD8^+^ infiltration, making it a more promising candidate for ICIs. The use of spartalizumab (anti–PD-1) in a phase I/II trial (NCT0240441) showed an ORR of 19% in ATC, with some durable responses (95). However, most patients developed rapid disease progression, underscoring the need for rational combination strategies.

### 5.2 Combination approaches to enhance ICI efficacy

To overcome limited monotherapy responses, combination immunotherapies are under active investigation. Clinical trials test dual immunotherapy, like combining PD-1 and CTLA-4 inhibitors (NCT03246958). Combining immunotherapy with tyrosine kinase inhibitors (TKIs) may enhance effectiveness in ATC. Combining pembrolizumab and TKIs improves survival in patients with ATCs expressing PD-L1 (106). Further, an anti-PD-1/PD-L1 monoclonal antibody combined with a BRAF inhibitor minimized tumor size in a mouse model of ATC and prolonged survival time (107). Additional phase II trials are investigating the combination of multikinase inhibitors (MKIs) with immune therapy (NCT04521348, NCT06848920). In contrast to CAR-T cell therapy alone, CAR-T cell treatment against intercellular adhesion molecule-1 (ICAM-1) combined with PD-1 blockade in ATC suppressed tumor growth and improved survival (108). Clinical study of NKG2D CAR-NK in combination with PD-1 monoclonal antibody for ATC also ongoing (NCT06856278). A study tested the combination of RAI and durvalumab in recurrent/metastatic TC patients, finding promising results in some patients (109). Despite the disappointing results of a phase II study combining pembrolizumab with chemoradiotherapy for ATC (110), other combination strategies involving ICIs with radiotherapy and chemotherapy are currently being investigated (NCT05659186, NCT04675710). Oncolytic viruses (OVs) have the advantage of converting “cold” tumors into inflamed ones; indeed, trials are evaluating OV+PD-1 blockade synergy (111) (**Table 2**).

**Table 2:** Ongoing clinical trials investigating immune checkpoint inhibitors in thyroid cancer.

NCT ID	Study title	Indication	Intervention	Phase	Status	Study URL
NCT06970353	Phase II Trial of Tunlametinib in NRAS-Mutant Advanced Thyroid Cancer	RAIR-DTC	Tunlametinib + anti-PD-1 mAb	Phase II	Recruiting	https://clinicaltrials.gov/study/NCT06970353
NCT06856278	Clinical Study of NKG2D CAR-NK Combined with PD-1 Monoclonal Antibody in the Treatment of ATC	ATC	NKG2D CAR-NK + anti-PD-1 mAb	Phase I/II	Recruiting	https://clinicaltrials.gov/study/NCT06856278
NCT03246958	Nivolumab Plus Ipilimumab in Thyroid Cancer	TC	Nivolumab + Ipilimumab	Phase II	Completed	https://clinicaltrials.gov/study/NCT03246958
NCT05119296	Phase II Trial of Pembrolizumab in Metastatic or Locally Advanced Anaplastic/Undifferentiated Thyroid Cancer	Advanced Anaplastic or Undifferentiated TC	Pembrolizumab	Phase II	Active not recruiting	https://clinicaltrials.gov/study/NCT05119296
NCT06848920	Study of Sinibolimab Combined with Lenvatinib As Neoadjuvant Therapy for Locally Advanced Unresectable Differentiated Thyroid Carcinoma	DTC	Sinibolimab + Lenvatinib	Phase II	Recruiting	https://clinicaltrials.gov/study/NCT06848920
NCT05059470	IMRT Followed by Pembrolizumab in the Adjuvant Setting in Anaplastic Cancer of the Thyroid (IMPAACT): Phase II Trial Adjuvant Pembrolizumab After IMRT in ATC	ATC	IMRT+Pembrolizumab	Phase II	Active, not recruiting	https://clinicaltrials.gov/study/NCT05059470
NCT04675710	Pembrolizumab, Dabrafenib, and Trametinib Before Surgery for the Treatment of BRAF-Mutated Anaplastic Thyroid Cancer	ATC and Thyroid Gland Squamous Cell Carcinoma	Dabrafenib + Trametinib + Pembrolizumab ± Radiotherapy	Phase II	Active, not recruiting	https://clinicaltrials.gov/study/NCT04675710
NCT05696548	Nivolumab Plus Lenvatinib Against Anaplastic Thyroid Cancer (NAVIGATION)	ATC	Nivolumab + Lenvatinib	Phase II	Active, not recruiting	https://clinicaltrials.gov/study/NCT05696548
NCT05659186	PD-1 Inhibitor and Anlotinib Combined With Multimodal Radiotherapy in Recurrent or Metastatic Anaplastic Thyroid Cancer	Recurrent/Metastatic ATC	Tislelizumab + Anlotinib ± Radiotherapy	Phase II	Recruiting	https://clinicaltrials.gov/study/NCT05659186
NCT04521348	Multiple Target Kinase Inhibitor and Anti-Programmed Death-1 Antibody in Patients With Advanced Thyroid Cancer	Advanced TC	mTKI + anti-PD-1 mAb	Phase II	Active, not recruiting	https://clinicaltrials.gov/study/NCT04521348

ATC, anaplastic thyroid carcinoma; CAR-NK, chimeric antigen receptor-engineered natural killer cells; DTC, differentiated thyroid cancer; IMRT, intensity-modulated radiation therapy; mAb, monoclonal antibody; mTKI, multiple tyrosine kinase inhibitor; RAIR, radioiodine-refractory; TC, thyroid cancer.

### 5.3 Immune-related adverse events and safety considerations

Although ICIs have expanded therapeutic opportunities for advanced thyroid cancers, treatment-related toxicities remain an important clinical concern (112). Immune-related adverse events (irAEs) result from nonspecific immune activation and may affect virtually any organ system, including the endocrine glands, skin, gastrointestinal tract, liver, and lungs (112).

Among endocrine toxicities, thyroid dysfunction represents one of the most frequently observed irAEs during PD-1/PD-L1 blockade (61). Patients commonly develop transient thyrotoxicosis followed by hypothyroidism, reflecting immune-mediated thyroid destruction (61). Interestingly, several studies have suggested that the development of thyroid irAEs may correlate with improved anti-tumor responses, potentially serving as a surrogate marker of immune activation (113).

Patients with pre-existing autoimmune thyroid disease may represent a unique population during immunotherapy (12). Chronic lymphocytic thyroiditis is characterized by activated lymphocytic infiltration and elevated immune checkpoint expression, raising the possibility that these tumors may exhibit enhanced sensitivity to PD-1/PD-L1 blockade (13). However, the coexistence of autoimmune disease may also increase the risk of endocrine irAEs and warrants careful monitoring (114).

Current evidence suggests that most endocrine irAEs can be effectively managed through hormone replacement and multidisciplinary surveillance without requiring permanent discontinuation of immunotherapy (112). Nevertheless, severe toxicities involving myocarditis, pneumonitis, or neurologic complications remain uncommon but potentially life-threatening (112). Future studies are needed to identify predictive biomarkers that can distinguish patients likely to benefit from immunotherapy from those at increased risk of serious toxicity (115).

### 5.4 Emerging immunotherapies

Adoptive Cell Therapies: Efforts are underway to engineer CAR-T cells targeting specific thyroid cancer antigens. In thyroid cancer, CAR-T therapy is at the preclinical/early clinical stage. Multiple thyroid-associated targets have been explored *in vitro* and animal models. Preclinical CAR-T constructs against TSH receptor (TSHR), ICAM-1, GFRα4, B7-H3, and CEA have shown thyroid cancer cell lysis (116). For example, TSHR CAR-Ts killed PTC cell lines in mice, and CEA CAR-Ts target MTC cells.

Clinically, early trials are underway. Notably, a Phase I trial of ICAM-1–directed CAR-T cells is recruiting patients with relapsed/refractory poorly differentiated or anaplastic thyroid carcinoma (117). Another approach (GAL-3 CAR-T, also known as AIC100) is in development for advanced thyroid cancer, as reported (98, 99). While CAR-T therapy has revolutionized hematologic cancers, solid tumors like TC present challenges: heterogeneous antigen expression, an immunosuppressive TME, and limited T cell infiltration. Thyroid CAR-T researchers are addressing these via optimized CAR design (e.g. dual-target CARs) and combination with checkpoint blockade. At present, data on clinical efficacy are pending. Overall, CAR-Ts offer a promising but experimental approach, potentially suited for highly antigenic TC subtypes (e.g. BRAF-wide type ATC or MTC).

Cancer Vaccines and Personalized Neoantigen Platforms: Discovering specific antigens in thyroid tumors could lead to the development of a vaccine targeting dendritic cells and T cells for TC. Proteins like thyroglobulin and thyroid peroxidase, along with tumor-associated antigens (TAA) such as MAGE, MUC1, and c-MET, may be expressed in DTC (100, 118, 119). Studies have found higher genetic alterations and thyroid tumor neo-antigen burden in poorly differentiated and anaplastic tumors compared to early stages (120). DC vaccines targeting cancer/testis antigen (CEA) have shown promise in treating these patients with medullary thyroid cancer (MTC) (121). New York esophageal squamous carcinoma 1(NY-ESO-1) is an important target for tumor vaccine immunotherapy, and treating BCPAP cells with 5-aza-2’-deoxycytidine can increase its expression, showing that TCR-based immunotherapies can induce antigen expression in TC cells (122).

**Figure 4:**
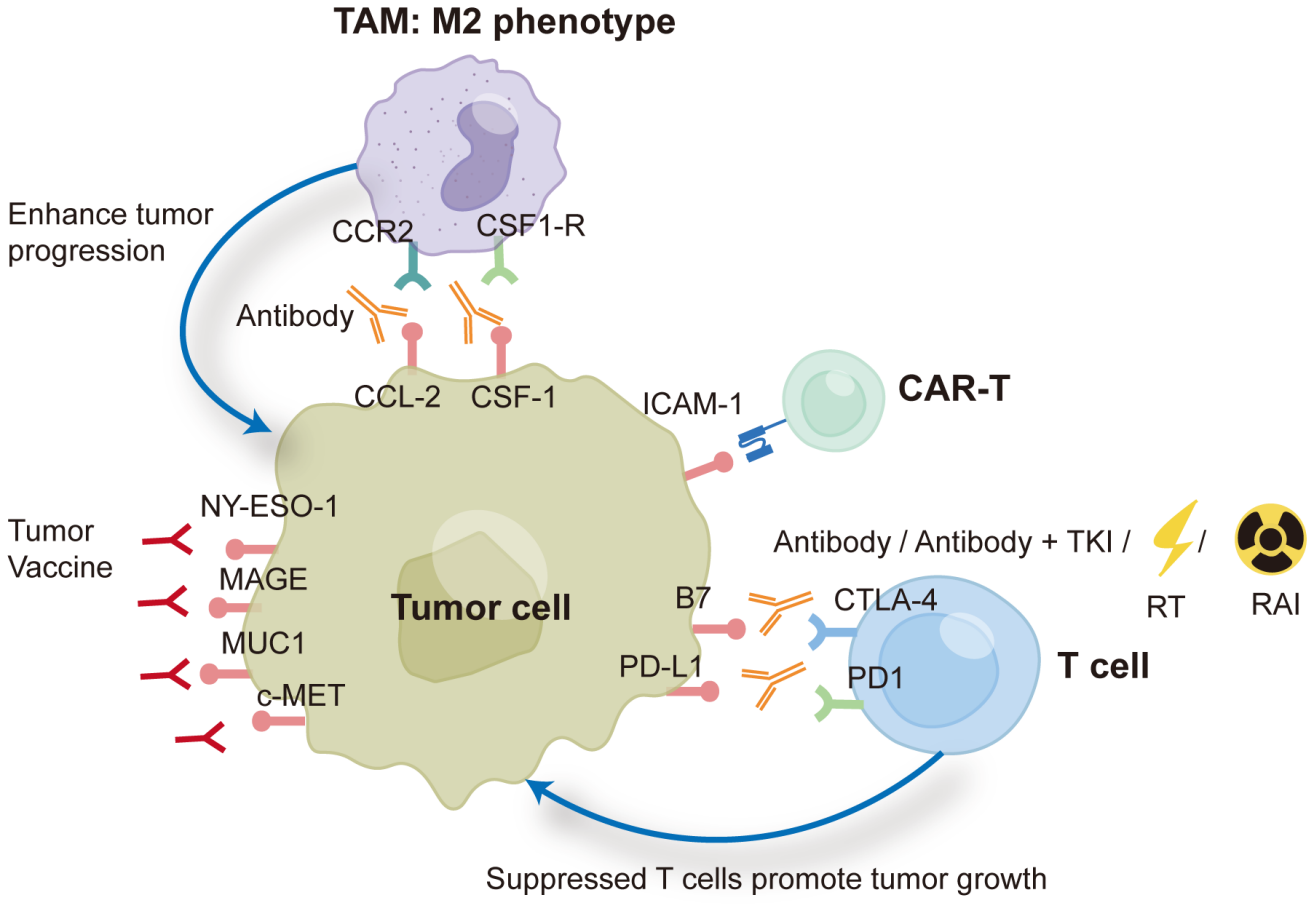
Emerging Immune treatment for thyroid cancer. Strategies such as tumor vaccine therapy, immune checkpoint inhibitor (ICI) therapy, ICI combined with TKI/IR/RAI therapies, TAM inhibition therapy, CAR-T therapy, and key cellular and molecular markers are depicted. TAMs promote tumor growth, while suppressed T cells enhance tumor progression. TKI, Tyrosine kinase inhibitors; TAMs, tumor-associated macrophages; RT, radiotherapy; RAI, radioactive iodine.

Targeting Immunosuppressive Cells: Inhibiting Tregs, myeloid-derived suppressor cells (MDSCs), or tumor-associated macrophages (TAMs) can restore anti-tumor immunity. ICI therapy in cancer may be hindered by M2-type TAMs. Many therapies targeting macrophages aim to prevent immature myeloid cells from entering the tumor microenvironment or to convert TAMs into immune-stimulating M1-like macrophages. Increased levels of colony stimulating factor 1 (CSF-1) and CCL2 were found in TC tissues (64), which attract TAMs. Targeting CSF-1/CSF-1R and CCL-2/CCR2 pathways could be effective in treating TC. This approach aims to prevent the recruitment of pro-tumor M2 type TAMs and convert them into the anti-tumor M1 phenotype (**Figure 4**).

### 5.5 Special considerations in pediatric thyroid cancer

Pediatric thyroid cancer represents a biologically distinct entity from adult thyroid cancer and may exhibit unique immune microenvironmental characteristics (123). Although pediatric papillary thyroid carcinoma (PTC) often presents with a higher frequency of lymph node metastasis and more extensive disease at diagnosis, long-term survival remains excellent in most patients (123).

Recent molecular studies have demonstrated that pediatric thyroid cancers are enriched for gene fusion events, including RET and NTRK rearrangements, whereas adult tumors more frequently harbor BRAF mutations (124). These distinct genomic landscapes may influence immune cell recruitment and tumor-immune interactions (124). Compared with adult thyroid cancer, pediatric tumors often display increased immune cell infiltration and heightened immune signaling activity, suggesting a potentially more immunogenic tumor microenvironment (125).

Despite these observations, data regarding immunotherapy in pediatric thyroid cancer remain extremely limited (126). Most immune checkpoint inhibitor trials have focused on adult populations, and evidence supporting their use in children with thyroid malignancies is largely extrapolated from adult studies (127). Given the excellent overall prognosis of most pediatric patients, the potential benefits of immunotherapy must be carefully balanced against long-term treatment-related toxicities and endocrine sequelae (123, 126).

Future studies integrating genomic, immunologic, and spatial profiling technologies may help identify pediatric patients with aggressive disease who could benefit from immune-based therapeutic strategies (57). International collaborative studies will be essential for defining the role of immunotherapy in this rare patient population.

### 5.6 Current limitations and future directions

Current understanding of thyroid cancer immunology remains limited by several important factors. First, most available studies are retrospective and involve relatively small patient cohorts, resulting in substantial heterogeneity across reported findings. Second, differences in sample processing, immune-cell annotation strategies, and biomarker assessment methods hinder cross-study comparisons and reproducibility. Third, many proposed biomarkers, including PD-L1 expression, IFN-γ signatures, and immune-cell infiltration scores, have not yet undergone prospective multicenter validation in thyroid cancer. In addition, most mechanistic insights are derived from bulk transcriptomic analyses, which may obscure spatial and functional heterogeneity within the tumor microenvironment. Although recent advances in single-cell and spatial technologies have begun to address these limitations, their clinical implementation remains in its early stages. Future studies should prioritize standardized immune profiling, multicenter prospective cohorts, and integrated spatial multi-omics approaches to establish clinically actionable biomarkers and optimize patient selection for immunotherapy.

## 6 Discussion

The relationship between TC and the immune system is complex and incompletely understood. Immune cells within the tumor microenvironment exert diverse and sometimes opposing effects on tumor progression and therapy response. This review integrates current transcriptomic and immunophenotypic evidence to characterize thyroid tumors into distinct immune phenotypes (hot, altered, and cold), providing a valuable framework for precision immunotherapy.

Immunotherapeutic approaches need to consider the immune status of TC. For tumors with an activated immune system (e.g., ATC-like tumors), restoring the anti-tumor immune response involves clearing immunosuppressive signals and depleting immunosuppressive cells. In contrast, for tumors with a non-activated immune system (e.g., PDTC-like tumors), including strategies like radiotherapy, chemotherapy, and targeted therapies can activate immune cells to induce tumor cell death.

Understanding why tumors exhibit different immune microenvironments and how T cells are recruited and activated in the TME is crucial for developing personalized TC treatment regimens. Additionally, optimizing the use of immunotherapy in advanced thyroid cancer, such as determining the timing of new treatments for RAIR-DTC, requires further research, including well-designed clinical trials to assess treatment effectiveness and balance survival extension with maintaining quality of life. Identifying the most effective treatment strategies to minimize resistance and ineffective drug exposure is paramount in advanced thyroid cancer management.
